# Filamentous Fungi Are Potential Bioremediation Agents of Semi-Synthetic Textile Waste

**DOI:** 10.3390/jof9060661

**Published:** 2023-06-13

**Authors:** Rachel Harper, Suzy Clare Moody

**Affiliations:** School of Life Sciences, Pharmacy, and Chemistry, Kingston University, Kingston upon Thames KT1 2EE, UK; r.harper@kingston.ac.uk

**Keywords:** mycoremediation, bioremediation, textile, dye, white rot, brown rot, *Hypholoma*

## Abstract

Textile waste contributes to the pollution of both terrestrial and aquatic ecosystems. While natural textile fibres are known to be biodegraded by microbes, the vast majority of textiles now contain a mixture of processed plant-derived polymers and synthetic materials generated from petroleum and are commonly dyed with azo dyes. This presents a complex recycling problem as the separation of threads and removal of dye are challenging and costly. As a result, the majority of textile waste is sent to landfill or incinerated. This project sought to assess the potential of fungal bioremediation of textile-based dye as a step towards sustainable and environmentally-friendly means of disposal of textile waste. Successful development of an agar-independent microcosm enabled the assessment of the ability of two fungal species to grow on a range of textiles containing an increasing percentage of elastane. The white rot fungus *Hypholoma fasciculare* was shown to grow well on semi-synthetic textiles, and for the first time, bioremediation of dye from textiles was demonstrated. Volatile analysis enabled preliminary assessment of the safety profile of this process and showed that industrial scale-up may require consideration of volatile capture in the design process. This study is the first to address the potential of fungi as bioremediation agents for solid textile waste, and the results suggest this is an avenue worthy of further exploration.

## 1. Introduction

The textile industry is a major source of pollution throughout the lifecycle of a garment, from the use of fossil fuels for the production of common synthetic textiles [1] to water contamination from dyes [2], from microplastic generation through washing [3] to air, terrestrial and aquatic pollution from landfill and incineration [4]. Textile waste presents a complex disposal problem due to the heterogeneity of composition, the volume of waste [5], and the disproportionate concentration of both production and disposal in low- and middle-income countries (LMICs) [6]. Most garments are now at least in part, synthetic, whether this is from the thread for seams, elastane to give stretchiness, plastic buttons or zips, or synthetic fibres such as nylon and polyester. While natural fibres such as cotton and wool are known to biodegrade over time [7,8], this can be negatively impacted by dyes and other processing and the end result is the prolonged presence of processed natural microfibres and synthetic microfibres in the environment [9,10]. As both landfill and incineration are unsustainable, a better way of processing the large volume of textile waste currently generated must be found.

**Figure 1:**
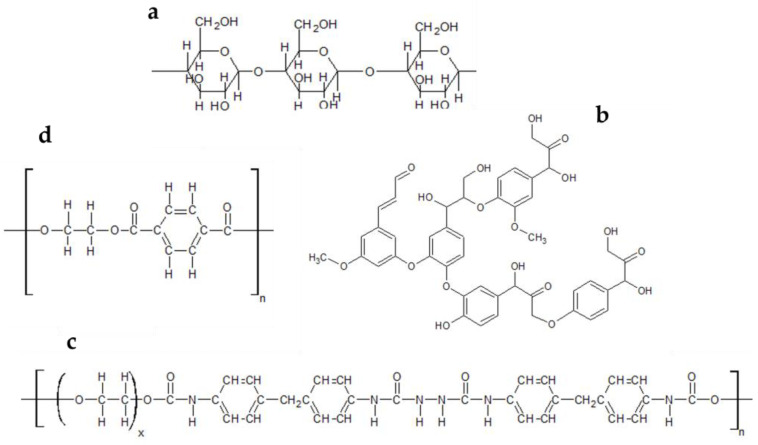
Indicative structures of (clockwise from the top): (**a**) cellulose, (**b**) lignin, (**c**) elastane, (**d**) polyester. Note the commonality of aromatic rings and carbon to oxygen bonding patterns in both natural and petroleum-derived materials.

The use of biological agents to eliminate polluting substances [11] is a well-established field, with both fungi and fungal enzymes already being investigated for use in the bioremediation of radioactivity, chemical spills, and microplastics [12,13,14,15]. Filamentous fungi are so named for their hyphal growth which enables exploration of the ecological niche and capture of nutrient resources. The hyphal morphology suggests this group of fungi as candidates for bioremediation of solid textile waste as they could potentially grow on and through the textiles. Further to this a specific group of filamentous fungi, the wood decay fungi, potentially have the enzymatic capacity to utilize the various components of semi-synthetic textiles. The wood decay fungi are ecological specialists adapted to perform lignocellulose degradation through the action of a comprehensive range of extracellular enzymes. Lignin is a polyphenolic compound of irregular structure that is highly recalcitrant to breakdown and, combined with the polymeric chains of cellulose and hemi-cellulose, forms lignocellulose in plants. Commonly used textiles include cotton (predominantly cellulose), viscose (a highly processed form of cellulose), and petroleum-derived synthetics such as polyester and elastane (aromatic polymers with some structural similarity to lignin) (see Figure 1).

Two broad mechanisms of wood decay, brown and white rot, have been described as opposite ends of a spectrum of activity. White rot fungi are capable of complete mineralization of lignocellulose through the action of low-specificity peroxidases, laccases, cellulases, and other carbohydrate-active enzymes (cazymes), reducing complex heteropolymers in the environment into smaller compounds which can be assimilated to support growth [16]. Brown rot fungi alter lignin without utilizing it via the oxidative Fenton reaction to enable the capture of the cellulose component of woody resources through cazymes [16]. Both mechanisms are non-specific and extracellular [17] suggesting they may be adapted to textile bioremediation.

Previous works, dating back almost a century, have identified various fungi that can degrade natural fibres such as cotton and wool, although these were conducted over short timescales and did not demonstrate complete bioremediation [7,8,18]. Likewise, initial steps of nylon degradation by white rot fungi have also been documented in laboratory conditions, using agar plates and sterile nylon discs as growth media [19,20]. Dye biodegradation has been the subject of many studies, in the context of wastewater treatment. Azo dyes, such as Reactive Black 5 (RB5) used in this study, are widely used but known to be toxic, mutagenic, and carcinogenic in waste [21]. This background, combined with the ecology and enzymatic capacity of wood decay fungi, suggested they had unexploited bioremediation potential. The hypothesis underpinning this research was that wood decay fungi would be able to grow on semi-synthetic textiles, with both white rot and brown rot fungi as potential candidates. The trial textiles used contained cotton, bamboo viscose, and an increasing percentage of elastane, and were dyed with RB5. The objectives were (i) to develop an agar-free microcosm that would be scalable for industrial use; (ii) to assess the ability of the fungi to bioremediate a commonly used dye from the textile; (iii) to analyze the volatile metabolome released during growth as a preliminary safety assessment of the process. The results of the study suggest wood decay fungi are worth further investigation as textile bioremediation agents.

## 2. Materials and Methods

### 2.1. Fungal Culture and Colonization of Woodchips

The strains used in this study were *Serpula himantioides* (MUCL38935, from the Belgian Co-ordinated Collections of Microorganisms) and *Hypholoma fasciculare* (HfGTWV2, from Professor Lynne Boddy at Cardiff University, Wales). Fungal strains were maintained on 2% malt extract agar (MEA, malt extract Oxoid, UK, agar Thermo Fisher Scientific, Waltham, MA, USA). 2 cm^3^ blocks of Scots Pine (*Pinus sylvestris*, sourced from Portswood Timber Supplies, Southampton, UK) and European Beech (*Fagus sylvatica*, sourced from John Harrison, Wrexham, UK) were chipped using hammer and chisel, then autoclaved and colonized as in [22] with *S. himantioides* and *H. fasciculare* respectively. These were incubated at 25 °C for 4 weeks in the dark, to allow the fungi to fully colonize the wood chips before they were added to the microcosm setup.

### 2.2. Microcosm Setup

**Figure 2:**
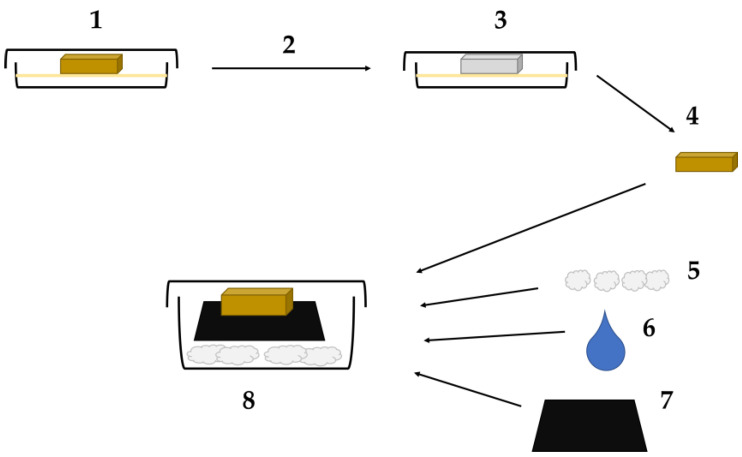
(**1**) the fungi were inoculated onto malt extract agar with wood chips. (**2**) the cultures were incubated at 25 °C for 4 weeks in the dark. (**3**) the fungus colonized the wood chips. (**4**) the colonized wood chip was removed from the culture and the excess agar scraped off. (**5**) sterile perlite added to polypropylene container. (**6**) 15 mL sterile water added to perlite. (**7**) 3 squares of pre-washed trial textile added. (**8**) scraped colonized wood block placed on top of trial textile. The microcosm was incubated in the dark at 25 °C.

A layer of sterile perlite (Westland Horticulture Ltd., Dungannon, UK) was placed in the bottom of each sterile 250 mL polypropylene pot, and approximately 15 mL of sterile dH_2_O was added. Using a sterile needle, 5 holes were poked in the side of each pot and were then covered with micropore tape (Wilko, Worksop, UK) to allow airflow. The three trial textiles were sourced from Bamboo Clothing Ltd. All contained the same plant-derived yarn—a blend of 70% bamboo viscose and 30% cotton—with either 0%, 4%, or 12% elastane, and all were black as a result of being dyed with Reactive Black 5 (RB5). The trial textiles were washed in a domestic washing machine using a non-biological washing powder 5 times at 30 °C with a 1000 rpm spin cycle, and line dried outside in between each wash. Three pieces of 3 cm × 3 cm textile were added to each pot. Pre-colonised wood chips were scraped to remove agar, then placed on top of the textile, in the centre of the pot. All pots were sealed, labelled, and incubated (LMS Cooled Incubator Model 600, LMS, Sevenoaks, UK) in the dark at 25 °C. They were checked regularly for water content and 5 mL sterile dH_2_O added to the perlite if the fabric felt dry. The set up process is summarized in Figure 2.

### 2.3. Imaging

At certain time points, the textiles were imaged using scanning electron microscopy (SEM, Zeiss Evo 50, Zeiss, Germany). The time points captured the colonization of the textile by the fungus (3 months), a mid-way time point (5 months), and the end of the trial (8 months). Textile samples were affixed to titanium stubs and then placed into a sputter coater (Polaron SC7640 Sputter Coater, Quorum Technologies, Laughton, UK). One side of the textile sample was coated with gold/palladium, with the machine set to 2 kV for 2 min at 20 mA, with Argon pumped into the chamber and maintained at a pressure of 10^−7^ mbar/PA. Each sample was placed into the chamber of the SEM, with the beam set to 5 kV. Images were taken at various magnifications, ranging from 35 to 2500.

### 2.4. Determination of Dye Content

#### 2.4.1. Thermogravimetric Analysis

10 mg of textile was weighed into an alumina crucible for loading into a Thermogravimetric Analyzer (Mettler Toledo, Leicester, UK). The starting temperature was set to 25 °C, and the machine was programmed to increase to 1000 °C in increments of 10 °C per minute. Results were recorded as graphs recording mass change over time in the STARe software V10.00 (Mettler Toledo, Leicester, UK). Reactive Black 5 (RB5, Merck, Germany) was used as a control.

#### 2.4.2. Dye Extraction Essay

The method developed by [23] was used to assay the dye content of the textile. Textile samples with no fungi on them were used as a control. RB5 dye was used as a standard, with a range from 1 mg/L to 10 mg/L. Prior to taking absorbance measurements, the samples were all diluted 1:4 in 1.5% (*v*/*v*) aqueous sodium hydroxide, as the undiluted samples exceeded an absorbance reading of 1. Samples were measured on a Unicam Helios Epsilon spectrophotometer (Thermo Fisher Scientific, Waltham, MA, USA).

### 2.5. Gas Chromatography Mass Spectrometry of Volatiles

A StableFlex™ 2 cm SPME fiber (Supelco, St. Louis, MO, USA) was used to capture volatiles released from an opened microcosm for one hour before injection into an Agilent 6890N GC with 5973N Inert MSD and 7683 Injector (Agilent, Santa Clara, CA, USA). The GCMS methodology started at 80 °C and ramped to a maximum of 320 °C with the temperature increasing at a rate of 10 °C per minute—for a total run time of 29 min. Controls with the different textiles and the relevant woods were used and any peak found in these controls was eliminated from further analysis. Peak data was collected from Total Ion Chromatograms (TICs) generated using Chemstation (Agilent, Santa Clara, CA, USA), and potential compound matches were collected from the NIST 2.0 database (National Institute of Standards and Technology, Gaithersburg, MD, USA) for each peak. Quality cutoff point was 60, and only compounds that were at or above 60 were recorded. Controls of textile and wood were also analysed, with compounds present in controls being removed from the experimental data sets. Compounds were then manually curated using the following databases to identify any known functions or health hazards associated with them: MetaCyc Metabolic Pathway Database version 20 [24]; PhytoHub version 1.4 [25]; the Yeast Metabolome Database version 2.0 [26,27]; Golm Metabolome Database [28]; the Metabolomics Workbench (https://www.metabolomicsworkbench.org/search/index.php, accessed between May and June 2022) [29]; and PubChem [30] for hazards and toxicity.

### 2.6. Bioinformatic Analysis of Genomes

Genomic data were obtained from the Joint Genomes Initiative (JGI) website (Home—*Serpula himantioides* (*S. lacrymans* var shastensis) MUCL38935 v1.0 (doe.gov) and Home—*Hypholoma sublateritium* v1.0 (doe.gov)) [31] using the standard annotations and search terms ‘laccase’ and ‘peroxidase’. All results were included in the analysis.

## 3. Results

### 3.1. Fungal Growth on Textiles

**Figure 3:**
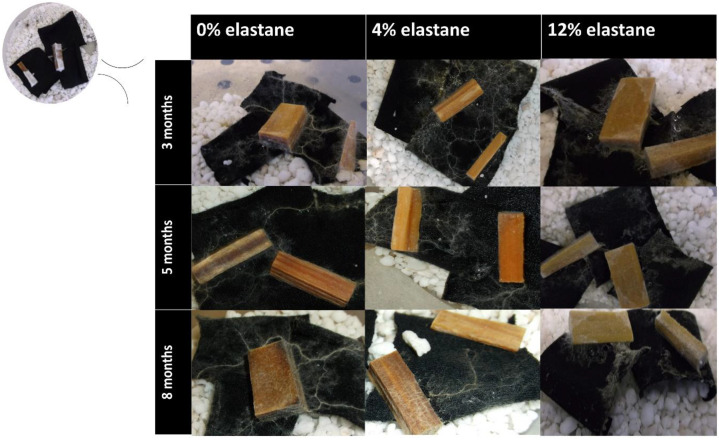
*S. himantioides* growing on a range of textiles, imaged at (top right, starting point) then at 3 months, 5 months, and 8 months incubation.

Neither fungal species used in this project had previously shown the ability to grow on textiles. Wood chips pre-colonized with either the white rot fungus *Hypholoma fasciculare* (HfGTWV2) or brown rot fungus *Serpula himantioides* (MUCL38935) were placed on textile and incubated in the dark at 25 °C for 8 months. The *S. himantioides* microcosms did not show any discolouration (Figure 3). The growth exhibited on all textiles was patchy, with some thin cord formation but predominantly a mycelial network that appeared to weave through the textile at times. This growth pattern has been previously seen in *S. himantioides* when grown on soil with wood resource units added (Skrede, unpublished), suggesting that the microcosm set up here provides similar nutrient options and foraging patterns to soil with sparse lignocellulose availability.

**Figure 4:**
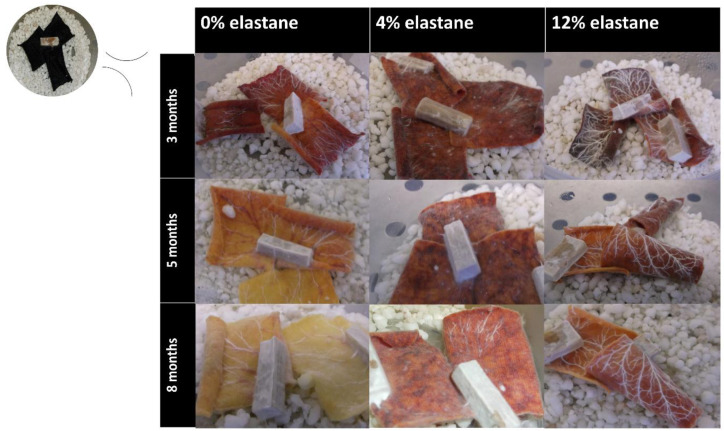
*H. fasciculare* growing on a range of textiles, imaged at (top right, starting point) then at 3 months, 5 months, and 8 months incubation.

**Figure 6:**
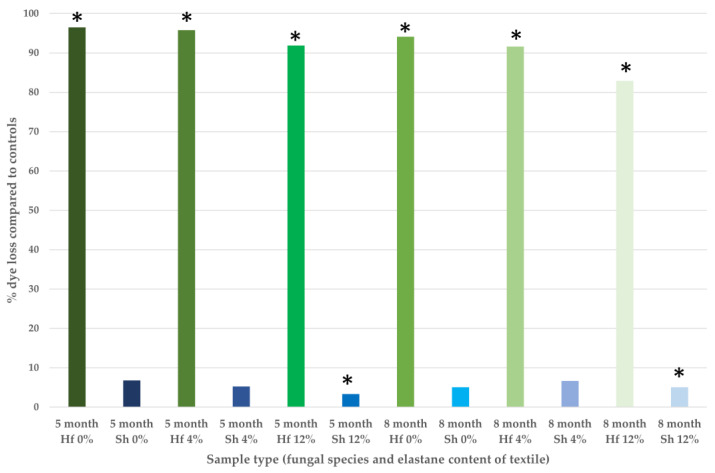
The dye loss from textiles at 5 and 8 months incubation with either *H. fasciculare* (Hf, shown in green) or *S. himantioides* (Sh, shown in blue), compared with the original textile. Percentages refer to elastane content. Each column shows the mean of three triplicate microcosms. The stars indicate samples that were significantly different from the control (as determined by paired two-tailed *t*-test).

Within 3 months, both species had grown across the textile but the *H. fasciculare* microcosms also exhibited a marked colour change. This grew more pronounced over time, and interestingly, was more obvious on 0% elastane textiles compared with both the 4% and 12% textiles (Figure 4). The growth pattern shown here is typical of all the microcosms, and the dense concentric rings produced by tangential anastomoses of mycelial growth seen in the agar culture of *H. fasciculare* were not produced on textile. Rather, the growth on all textiles closely echoed that described by [32] as the textile appeared to be thoroughly colonized with mycelia, with thick connecting cords extending across the textile resource from the wood inoculum. The presence of these foraging cords as a hyphal network were particularly noticeable on the 12% elastane textiles (Figure 4). As shown below, the fungus was able to metabolize dye more easily from 0% and 4% elastane textiles (see Figure 6) and it is suggested that this pattern of increased cord development on 12% elastane may be due to more extensive resource capture as the textile was the key nutrient source in these micrososms, rather than the dye. As wood decay fungi access nutrition from solid organic resources, spatial capture is equivalent to nutrient capture [33], and *H. fasciculare* is well- adapted as a forest floor competitor to enact this rapidly. At no point did *H. fasciculare* extend foraging cords beyond the boundaries of the textile, suggesting adequate nutrition was available within the textile microcosm.

**Figure 5:**
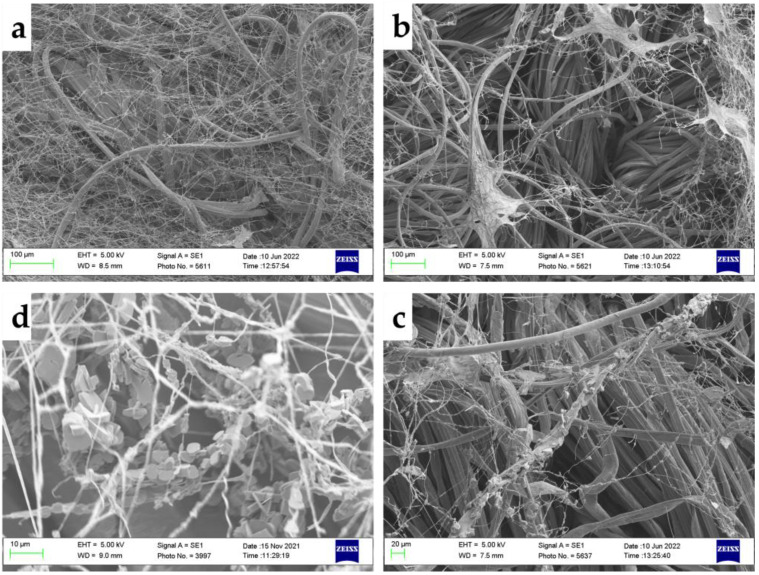
SEM images on a representative sample of microcosm textiles show the variety of structures seen. Clockwise from top left: (**a**) *H. fasciculare* on 12% elastane at 8 months incubation, (**b**) *S. himantioides* on 0% elastane at 8 months incubation, (**c**) *S. himantioides* on 4% elastane at 8 months incubation, (**d**) *H. fasciculare* on 4% elastane at 5 months incubation.

Scanning Electron Microscopy (SEM) imaging of the textiles and individual fibres was undertaken to give a baseline and means of identifying any fibre preference the fungi may have displayed. The images are included in the supplementary information (Figure S1). Imaging a sample of colonized textiles by SEM revealed hyphal growth on all fibre types, with denser growth in the *H. fasciculare* microcosms (Figure 5) which confirmed the light microscopy findings. The images shown are a representative sample showing exemplars of the different structures. Figure 5a,b,d were taken at 8 months, but Figure 5c is from 5 months as no crystals were detected in *H. fasciculare* samples at 8 months. The hyphal morphologies seen in both species appeared normal compared with others’ images [34], with some patchy structures (Figure 5b) likely to be fungal metabolites that have this sheet-like appearance under SEM conditions [35]. Crystalline structures (Figure 5c,d) were seen on all textiles at 3 months for both species. For *H. fasciculare*, they were also found on all three textiles at 5 months but were not visualized in the samples taken at 8 months. For *S. himantioides*, crystals were additionally seen on 0% and 4% at 5 months, and 4% and 12% at 8 months, suggesting that they may be present on all textiles at all time points for this fungus and increased sampling may have demonstrated this.

### 3.2. Quantification of Dye Remediation

Two methods of quantification were trialled. The unsuccessful method is reported here to enable others to benefit from the protocol development that contributed to this study.

#### 3.2.1. Thermogravimetric Analysis (TGA)

For TGA to be a useful method in this study each fibre type and the dye would need a significantly different thermal decomposition point, that would be detectable when plotted as mass change over time. The textile used was dyed with Reactive Black 5 (RB5), a common black clothing dye. The 0% elastane textile was compared with undyed bamboo viscose-cotton but little difference was noted. RB5 powder was analysed in isolation but rather than showing a distinct ‘step’ in mass loss, there was a gradual loss over time. Elastane alone was compared with both 0% and 12% textiles, but again, there were no distinct mass changes. These data are shown in Figure S2 and demonstrate that TGA was not a suitable method for assessing the remediation of the dye in this context.

#### 3.2.2. Dye Extraction and Spectrophotometric Quantification

The method that gave reproducible results was based on a protocol by [23]. The incubated textiles were compared with control samples from the same garments. All experiments were done in triplicate and the data presented in Figure 6 are the means.

At both 5 months and 8 months of incubation, the samples from *H. fasciculare* demonstrated a significant decrease in dye saturation as had been noted on visual examination of the microcosms. This was evident in all textile types with over 80% dye lost, although the 12% elastane textile showed a slightly lower level of dye remediation than the 4% and 0% textiles. The *S. himantioides* microcosm textiles were visually unchanged from the black starting point, but dye extraction and quantification showed that the 12% textile at 5 and 8 months incubation had a small but significant (*p*-value 0.001) drop in dye content compared with the controls.

### 3.3. Bioinformatic Analysis of Enzyme Capacity for Dye Remediation

**Figure 7:**
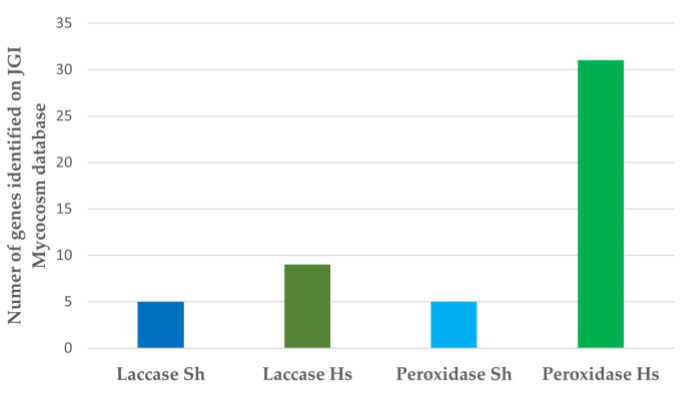
The laccase and peroxidase complement of *S. himantioides* (Sh, shown in blue) and *H. sublaterium* (Hs, shown in green). Data were obtained from the Joint Genomes Initiative website (Home—*Serpula himantioides* (*S. lacrymans* var shastensis) MUCL38935 v1.0 (doe.gov) and Home—*Hypholoma sublateritium* v1.0 (doe.gov)) [31].

Bioinformatic analysis of enzyme candidates for dye remediation offered a route to understanding the disparity in dye loss from the textiles between the two fungal species. The genome for *H. fasciculare* was unavailable at the time of writing, but that of a close relative, *Hypholoma sublaterium*, was used as a proxy to compare the potential enzymatic degradative capability of the two fungal species used. Two groups of key secreted enzymes, the laccases and peroxidases, known to be involved in the breakdown of complex phenolic compounds as part of the wood decay ecology, were analyzed. As seen in Figure 7, the *Hypholoma* genus appears to have far greater numbers of laccases and peroxidases which may account for the disparity in dye remediation seen here. It is acknowledged that many other enzymes may also be involved in this process and that *H. fasciculare* may differ from *H. sublaterium* in enzyme complement.

### 3.4. Gas Chromatography Mass Spectrometry (GCMS) Analysis of the Volatile Metabolome

The volatile metabolome was captured and analysed by GCMS at three timepoints during the study, with uncolonized wood and textile used as controls to exclude volatiles released from the substrates. The GCMS spectra showed a change in volatile profile between the fungi, between different textiles, and over time. The majority of the compounds identified had no previously published connection to fungal metabolomics which made interpretation of function or role challenging (tables of tentatively identified compounds are included in Table S1). Metabolites of interest with identified function in at least two replicates of both fungal microcosms were butylated hydroxytoluene (a known lipophilic fungal metabolite with antioxidant properties) and dodecane (a medium chain alkane with known involvement in the biosynthesis of cuticular wax in vascular plants). Phytane (a diterpenoid specialized metabolite) was identified in at least two *H. fasciculare* 4% elastane microcosms, with 1,5,9-trimethyl-1,5,9-cyclododecatriene (a known plant volatile) released in at least two of the *S. himantioides* 4% elastane microcosms. Farnesane, a sesquiterpenoid specialized metabolite, was also identified in at least one microcosm of each fungus, suggesting that terpenoid metabolism may play a role in dye metabolism or textile degradation. There were different timescales and longevity for the production of these metabolites. For example, butylated hydroxytoluene was seen in *H. fasciculare* at 3 months on 4% elastane, whereas dodecane was seen in *H. fasciculare* at both 3 and 5 months incubation but only on 12% elastane. Butylated hydroxytoluene was detected in *S. himantioides* microcosms on all three textiles at 3 months incubation, but dodecane was only detected on 4% elastane at 3 months. The variability of the volatile metabolome over time and between samples was noted, being particularly pertinent at 8 months of incubation. At this time point, although volatiles were produced, no compounds were identified in more than one biological replicate in any of the conditions for either fungus. This high level of variation was not entirely unexpected given the few volatiles consistently identified in more than one replicate at earlier time points but is certainly an interesting avenue to explore further to understand why this variation may exist.

## 4. Discussion

The number of textile items produced globally has doubled in 15 years to more than 100 billion units, and when discarded, 73% of items are incinerated or sent to landfill [36]. As at least 60% of clothing contains synthetic fibres [37], it is not surprising that the fashion industry causes significant environmental and societal damage throughout the lifecycle. New research into alternative sources for textiles and methods of disposal is urgently needed.

This project signifies the first demonstration of fungal growth on textiles with elastane, with the development of agar-free microcosms successfully maintaining fungal culture for over 6 months. There were clear differences noted in the extent of hyphal growth and particularly cord formation in *H. fasciculare* on the different textiles (Figure 4). It was suggested this may be due to differential nutrient metabolism based on the elastane content, and further work is needed to develop a method for quantification of the relative textile components (cotton, bamboo viscose, elastane, and RB5 dye). This would enable a greater understanding of which specific components of the microcosm resources are being utilized by the fungi over time. Development of a protein extraction protocol for this microcosm set up has proved challenging, but successful capture of the proteome would enable important insights into the processes and metabolic pathways the fungi are using to maintain growth on the different textiles, and potentially provide greater knowledge of preferential resource use.

As far as the authors are aware, this is also the first demonstration of dye bioremediation directly from textiles by any fungus. This was not unexpected as other white rot fungi have previously been shown to be capable of RB5 degradation (e.g., *Trametes hirsuta* [38]) but in solution, rather than from textile. The results shown here demonstrate the biodegradation of the dye rather than the adsorption reported by others [21] as the hyphae remained white throughout the experiment (Figure 3 and Figure 4). *H. fasciculare* demonstrated some impact of elastane content on dye removal as the 12% textile contained more dye at the 8 month incubation point than the 4% or 0% textiles (Figure 6). It is unclear whether this is due to the different chemical interactions of the dye with natural and synthetic textiles having an impact on the bioavailability of the fungus to metabolise the dye. In contrast, the 12% elastane textile appears to facilitate *S. himantioides* ability to utilize the dye. At both 5 and 8 month timepoints, there is a small but significant decrease in the amount of dye remaining in the textile compared to the control (Figure 6). This may suggest a different mechanism or underlying process by which these two fungi approach dyed textiles, which would be in keeping with their differential ecologies.

While this project did not attempt identification of the crystals seen on SEM images of both fungi, they bear significant structural similarity to whewellite, calcium oxalate monohydrate, which has been previously shown to be a fungal metabolite of *S. himantioides* [39] and had a very similar arrangement on the hyphae compared with whewellite crystal production by *Perenniporia meridionalis* [40]. Calcium oxalate crystals are also known to be produced during an interaction between *H. fasciculare* and another fungus (although the producing organism was not identified in that study) and may be associated with a fungal stress response [41].

The volatile metabolome analyses suggested heterogeneous textile samples may be challenging for predictability and identification, but the presence of elastane while altering the volatiles produced did not cause a significant release of known hazardous volatiles during the lifespan of the incubations used here. The identified metabolites had few hazard symbols associated with them in PubChem [30] but some were designated as irritants or health hazards which may have implications for ensuring scaled up applications are appropriately managed. Similar to others, the degradation products produced in these microcosms were not easily characterized but their potential toxicity is likely to be low based on others work. White rot fungi have been shown to oxidise RB5, although it is thought that where the appearance of RB5 changes to a light red colour as it does in the microcosms in this work, this is indicative of the continued presence of metabolites containing azo bonds and naphthalene rings. In silico docking experiments have suggested laccases as being of paramount importance in the biodegradation of RB5 [38] although peroxidases have also been suggested as promising enzymes to explore [42]. Where a fungal biodegradation mechanism for RB5 has been elucidated and the end point metabolites captured, there has been a demonstration of limited toxicity to plant seeds [21,43] and reduced cytotoxicity to human cell lines [38].

The findings presented here are promising, but some significant challenges remain to be able to demonstrate mycoremediation as a sustainable option for industrial scale removal of textile pollution. The first is that long timescales, beyond the life span of many research funding options, would be needed to demonstrate complete degradation. It is currently unknown whether the synthetic materials in the textile would be metabolized, and if they were, it is not known what the breakdown products may be. It is possible that generation of microplastics would be the unwelcome endpoint. If mycoremediation can be demonstrated at a laboratory scale, the final challenge would be scaling this up to be a viable option at an industrial scale. While the experimental work described here has been designed with scale up in mind, there would still be challenges of increased production of volatiles (whether harmful or not), the increase in temperature in large composting facilities may impact fungal growth and metabolism, and the risk of contamination. Recognizing the possible challenges and realizing the potential of mycoremediation for textile waste remain a promising field for further exploration.

## Data Availability

We confirm we are happy to supply the original GCMS.RAW files if requested.

## Supplementary Materials

The following supporting information can be downloaded at: https://www.mdpi.com/article/10.3390/jof9060661/s1, Figure S1. SEM images of individual fibres to identify fibre types within the fabric. (a) shows a single woven thread of the bamboo viscose and cotton mix, with a total width of 230.7 µm. (b) shows individual fibres of cotton (left) and bamboo viscose (right) from the thread, with widths of 29.8 µm and 12.99 µm respectively. (c,e) show elastane fibres identified within the woven fabric structure seen in (g). (f) shows a clearer example of cotton vs. bamboo viscose fibres in the fabric, the bamboo viscose having striations and the cotton being less uniform. (h) shows some of the damage to the individual fibres seen in the washed fabric. These are representative images for the washed fabric controls, from several that were taken. Figure S2. Aggregate graph of thermogravimetric analysis (TGA) curves plotting the percentage of initial mass against temperature in °C for RB5 dye (black line), fabric with 12% elastane (red line), fabric with no elastane content (blue line) and the undyed bamboo viscose-cotton fibres (green line). However, no obvious difference in the decomposition of the 0% elastane fabric sample and the undyed bamboo viscose and the cotton mixture was noted, making it difficult to determine any dye loss via this method (blue and green lines). A sample of pure RB5 powder was analysed in the same way, and rather than showing a clear ‘step’ where a large mass loss occurred due to decomposition, it was more of a gradual loss over time. This confirmed that it would have been difficult to quantify dye loss via TGA (black line). Table S1. The volatile metabolome captured from microcosms at 3, 5, and 8-month timepoints on 0, 4, and 12% elastane fabrics. The NIST database was used for identification with a Quality Cutoff of 60. The metabolites included here were found in at least two biological replicates at that timepoint and on that fabric.
