# Supplementary material for: Filamentous Fungi Are Potential Bioremediation Agents of Semi-Synthetic Textile Waste
Source: J Fungi (Basel). 2023 Jun 13;9(6):661. doi: 10.3390/jof9060661 (PMC10301444; doi:10.3390/jof9060661)
Supplement: Supplementary file 1 [file jof-09-00661-s001.zip › jof-2406088-supplementary.pdf]

Fungal bioremediation of textiles: Supplementary Information

Figure S1. SEM images of individual fibres to identify fibre types within the fabric. (a) shows a single woven thread of the bamboo viscose and cotton mix, with a total width of 230.7 µm. (b) shows individual fibres of cotton (left) and bamboo viscose (right) from the thread, with widths of 29.8 µm and 12.99 µm respectively. (c) and (e) show elastane fibres identified within the woven fabric structure seen in (g). (f) shows a clearer example of cotton vs bamboo viscose fibres in the fabric, the bamboo viscose having striations and the cotton being less uniform. (h) shows some of the damage to the individual fibres seen in the washed fabric. These are representative images for the washed fabric controls, from several that were taken.

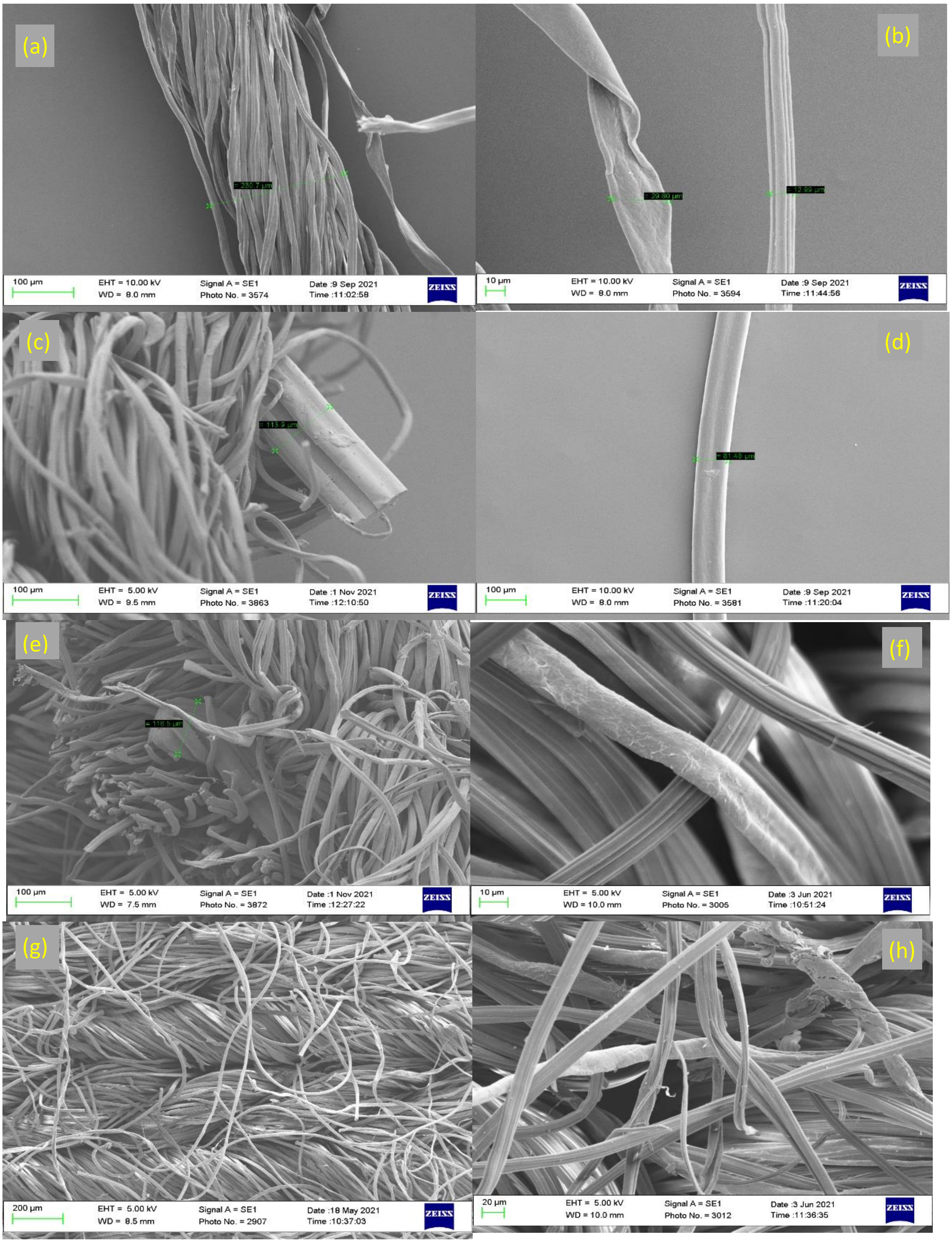
(a)
100 µm
EHT = 10.00 kV
WD = 8.0 mm
Signal A = SE1
Photo No. = 3574
Date :9 Sep 2021
Time :11:02:58
(b)
10 µm
EHT = 10.00 kV
WD = 8.0 mm
Signal A = SE1
Photo No. = 3594
Date :9 Sep 2021
Time :11:44:56
(c)
100 µm
EHT = 5.00 kV
WD = 9.5 mm
Signal A = SE1
Photo No. = 3863
Date :1 Nov 2021
Time :12:10:50
(d)
100 µm
EHT = 10.00 kV
WD = 8.0 mm
Signal A = SE1
Photo No. = 3581
Date :9 Sep 2021
Time :11:20:04
(e)
100 µm
EHT = 5.00 kV
WD = 7.5 mm
Signal A = SE1
Photo No. = 3872
Date :1 Nov 2021
Time :12:27:22
(f)
10 µm
EHT = 5.00 kV
WD = 10.0 mm
Signal A = SE1
Photo No. = 3005
Date :3 Jun 2021
Time :10:51:24
(g)
200 µm
EHT = 5.00 kV
WD = 8.5 mm
Signal A = SE1
Photo No. = 2907
Date :18 May 2021
Time :10:37:03
(h)
20 µm
EHT = 5.00 kV
WD = 10.0 mm
Signal A = SE1
Photo No. = 3012
Date :3 Jun 2021
Time :11:36:35

Figure S2. Aggregate graph of thermogravimetric analysis (TGA) curves plotting percentage of initial mass against temperature in °C for RB5 dye (black line), fabric with 12% elastane (red line), fabric with no elastane content (blue line) and the undyed bamboo viscose-cotton fibres (green line). However, no obvious difference in the decomposition of the 0% elastane fabric sample and the undyed bamboo viscose and cotton mixture was noted, making it difficult to determine any dye loss via this method (Figure 12, blue and green lines). A sample of pure RB5 powder was analysed in the same way, and rather than showing a clear 'step' where a large mass loss occurred due to decomposition, it was more of a gradual loss over time. This confirmed that it would have been difficult to quantify dye loss via TGA (Figure 12, black line).

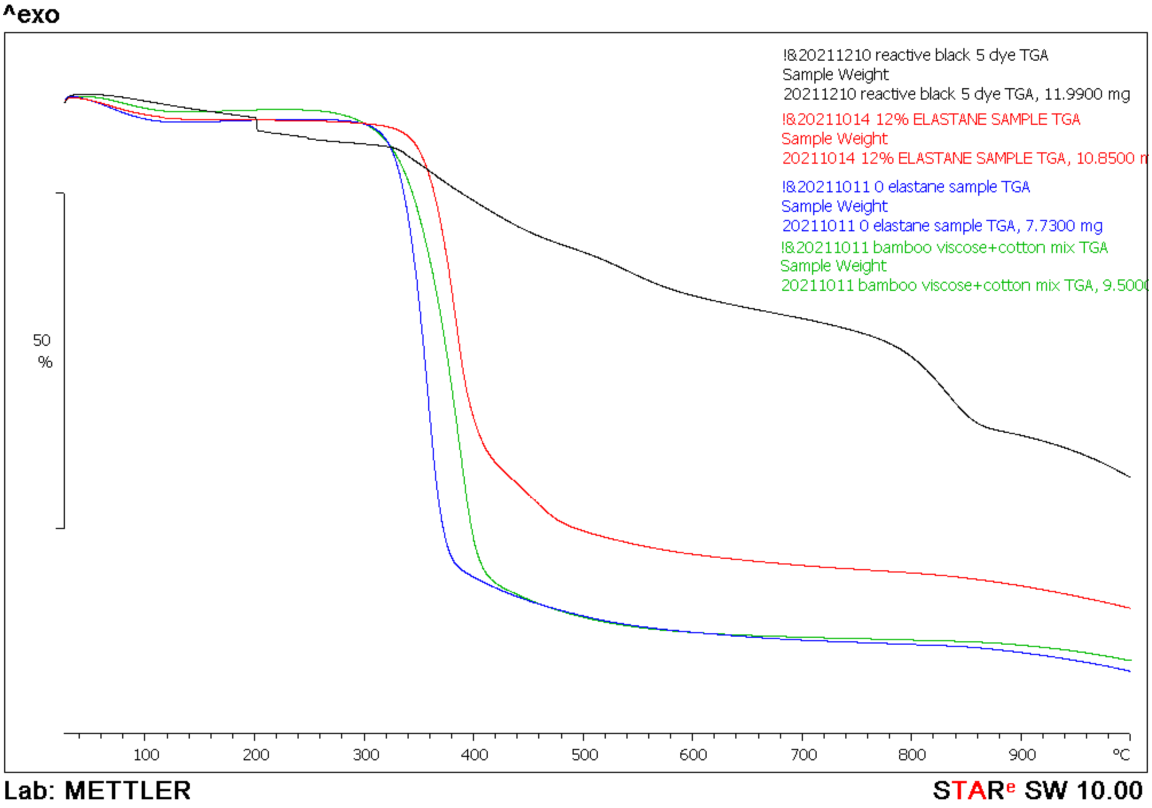

Table S1. The volatile metabolome captured from microcosms at 3, 5 and 8 month timepoints on 0, 4 and 12% elastane fabrics. The NIST database was used for identification with a Quality Cutoff of 60. The metabolites included here were found in at least two biological replicates at that timepoint and on that fabric.

**Number of compounds found in at least two replicates of *H. fasciculare* samples**			
Timepoint	0% elastane	4% elastane	12% elastane
3 months	0	Butylated Hydroxytoluene	4-Isopropyl-1,3-cyclohexanedione, dodecane
5 months	Nonane 4,5-dimethyl-, Undecane, Methoxyacetic acid 3-pentadecyl ester, 2H-2,4a-Methanonaphthalene 1,3,4,5,6,7-hexahydro-1,1,5,5-tetramethyl-, (2S)-,	Hexadecane 2,6,10,14-tetramethyl- (phytane),Heptadecane 8-methyl-, 2H-2,4a-Methanonaphthalene 1,3,4,5,6,7-hexahydro-1,1,5,5-tetramethyl- (2S)-	3,5-di-tert-Butyl-4-hydroxyacetophenone, 1,3,5-,
8 months	0	0	0
**Number of compounds found in at least two replicates of *S. himantioides* samples**			
Timepoint	0% elastane	4% elastane	12% elastane
3 months	1 - Butylated Hydroxytoluene	3 – Dodecane, butylated hydroxytoluene, 1,5,9-Cyclododecatriene, 1,5,9-trimethyl-	2 – dodecane, 2,6,10-trimethyl- (farnesane), butylated hydroxytoluene
5 months	6 - Eicosane, 3,5-di-tert-Butyl-4-hydroxyacetophenone, Propanamide N-(1-ethyl-1,2,3,4-tetrahydro-2,2,4-trimethyl-7-quinolinyl)-, 2,6-Bis(1,1-dimethylethyl)-4-(1-oxopropyl)phenol,	11 - Eicosane, Undecane, 10-Methylnonadecane, Hexadecane, Pentadecane, 2H-2,4a-Methanonaphthalene 1,3,4,5,6,7-hexahydro-1,1,5,5-tetramethyl-, (2S)-, Decane 3,6-dimethyl-, Phenol 2,6-bis(1,1-dimethylethyl)-4-(1-methylpropyl)-, 3,5-di-tert-Butyl-4-hydroxyacetophenone,	12- Eicosane, Nonadecane, Dodecane, 2H-2,4a-Methanonaphthalene 1,3,4,5,6,7-hexahydro-1,1,5,5-tetramethyl-, (2S)-, Dodecane 2-methyl-, Naphthalene 2-methyl-1-(3,4-dimethylbenzoyl)-, Decane 3,6-dimethyl-, Henicosane, Methoxyacetic acid 4-tetradecyl ester, 2H-2,4a-Methanonaphthalene 1,3,4,5,6,7-hexahydro-1,1,5,5-tetramethyl-, (2S)-,
8 months	0	0	0
